# Responding to the health needs of migrant farm workers in South Africa: Opportunities and challenges for sustainable community‐based responses

**DOI:** 10.1111/hsc.12840

**Published:** 2019-09-02

**Authors:** Thea de Gruchy

**Affiliations:** ^1^ The African Centre for Migration & Society University of the Witwatersrand Johannesburg South Africa

**Keywords:** community health workers, community‐based responses, migrant farm workers, migration and health, South Africa, sustainability

## Abstract

Reflecting global trends, migrant farm workers in South Africa experience challenges in accessing healthcare. On the commercial farms in Musina, a sub‐district bordering Zimbabwe, Medécins sans Frontières and the International Organization for Migration both implemented migration‐aware community‐based programmes that included the training of community‐based healthcare workers, to address these challenges. Using qualitative data, this paper explores the experiences that migrant farm workers, specifically those involved in the programmes, had of these interventions. A total of 79 semi‐structured interviews were completed with migrant farm workers, farm managers, NGO employees and civil servants between January 2017 and July 2018. These data were supplemented by a review of grey and published literature, as well as observation and field notes. Findings indicate that participants were primarily positive about the interventions. However, since the departure of both Medécins sans Frontières and the International Organization for Migration, community members have struggled to sustain the projects and the structural differences between the two programmes have created tensions. This paper highlights the ways in which local interventions that mobilise community members can improve the access that rural, migrant farming communities have to healthcare. However, it simultaneously points to the ways in which these interventions are unsustainable given the realities of non‐state interventions and the fragmented state approach to community‐based healthcare workers. The findings presented in this paper support global calls for the inclusion of migration and health in government policy making at all levels. However, findings also capture the limitations of community‐based interventions that do not recognise community‐based healthcare workers as social actors and fail to take into account their motivations, desires and need for continued supervision. As such, ensuring that the ways in which migration and health are included in policy making are sustainable emerges as a necessary element to be included in global calls.

**What is known about this topic**

- Increasingly calls are being made for migration aware policies and programming at all levels of governance.
- In South Africa, health policies do not consider the realities of migration, which affects the access that migrants, including migrant farm workers, have to care.
- South Africa's approach to community‐based interventions is fragmented and characterised by a reliance on non‐state actors.

**What this paper adds**

- Original, empirical insights into community‐based interventions, and their potential as migration‐aware responses to health.
- An analysis of why community‐based interventions are unsustainable in South Africa.
- Urgency to the call for sustainable migration aware policies and programmes.

## 1 BACKGROUND

Recently, calls have been made at a global level for policies, at all levels of government, to be migration and health aware (Khan et al., 2016; Vearey, 2014; Wickramage & Annunziata, 2018). Research from the South African context has shown that local responses—or a lack thereof—can contribute to either exacerbating or mitigating the experiences that migrants have of accessing healthcare (Halogen & Vearey, 2010; Landau & Singh, 2008; Misago, 2016; Vearey, 2011).

One kind of local, community‐based response to issues of health is programmes that train and equip community members to facilitate access to healthcare within the community. This approach has received renewed attention in South Africa, as well as in other low‐ and middle‐income countries (LMICs), since the mid‐1990s, as the under resourced South African public healthcare system is faced with multiple challenges in addressing the ‘four colliding epidemics’ of ‘HIV and tuberculosis; chronic illness and mental health; injury and violence; and maternal, neonatal, and child health’ (Mayosi et al., 2012; Nxumalo, Goudge, & Manderson, 2016; Schneider, Hlophe, & van Rensburg, 2008). However, although the state increasingly recognises the role that these programmes can play in addressing unmet health needs at the local level, policy responses have largely been fragmented and highlight that while the state sees these programmes and their cadres of workforce as important, they remain peripheral to the health system itself and their training and management is by‐and‐large left up to non‐governmental organisations (NGOs) (Daniels, Clarke, & Ringsberg, 2012; van Ginneken, Lewin, & Berridge, 2010; Schneider et al., 2008). This reliance on external non‐state actors has implications both for the sustainability of these programmes and for the security and well‐being of these workers (Clarke, Dick, & Lewin, 2008; Nxumalo et al., 2016; Suri, Gan, & Carpenter, 2007). Additionally, it has also meant that there is little uniformity in the structure of these programmes (Friedman, 2005; Schneider et al., 2008).

For the purposes of this paper, these individuals will be referred to as community‐based healthcare workers. However, within specific programmes they have different titles, varied responsibilities and levels of training, are sometimes volunteers and at other times are remunerated (Clarke et al., 2008; Friedman, 2005; Mwai et al., 2013).

This paper explores two programmatic interventions that independently developed cadres of community‐based healthcare workers in order to improve the access that migrant farm workers in the area around the South African town of Musina had to healthcare. Ten kilometres from the Zimbabwean border, Musina has always seen the coming and going of Zimbabwean nationals, some of whom have traditionally found work, both seasonally and more permanently, on the farms surrounding the town (Bolt, 2016; Rutherford, 2008; Rutherford & Addison, 2007). In 2007 and 2008, in response to an increase in the number of Zimbabweans crossing the border to escape electoral violence and a cholera outbreak in Zimbabwe (Staff Reporter, 2008; Tran, 2008), several international organisations, including the International Organization for Migration (IOM) and Médecins sans Frontières (MSF), set up projects in the area. By 2009, this crisis had largely dissipated, and both organisations moved their focus to migrant farm workers on the commercial farms surrounding Musina, as vulnerable groups whose access to healthcare was limited and could be improved.

Globally, while migrant farm workers are key to many commercial farming industries, more often than not structural barriers ensure that this workforce bears an undue burden of both communicable and non‐communicable diseases (Arcury & Quandt, 2007; Rye & Andrzejewska, 2010). South Africa is no exception. While legislation ‘covering rights to collective bargaining, basic conditions of employment, social security benefits and workplace health and safety’ (London, 2003, p. 60) exist, the access that migrant farm workers have to these rights, including to healthcare, is limited. Subject to low wages and poor living and working conditions (Bolt, 2012; Jinnah, 2017; London, 2003), within a context in which the public health system is severely under resourced and programming is not migration‐aware (Vearey, 2014, 2018; Vearey, Modisenyane, & Hunter‐Adams, 2017), migrant farm workers are known to have one of the highest HIV prevalence rates in the country (International Organization for Migration, 2010).

In Musina, at the time that MSF and the IOM were developing programmes, the Department of Health had a mobile clinic programme that was meant to visit farm worker communities on rotation. However, it was severely under resourced and, importantly, unable to provide any form of HIV care or support. Furthermore, continuity of care for HIV—and other chronic conditions—has historically been very difficult in this area as patients move regularly and South African health systems are yet to respond to the realities of patient mobility, both cross‐border and internal (Médecins Sans Frontières, 2012, 2013; Vearey, 2014; Vearey et al., 2017). To address these gaps, MSF implemented the Musina Model of Care, an initiative that included a mobile clinic programme that provided voluntary counselling and testing (VCT) for HIV, antiretroviral therapy (ART) and developed a cadre of Community Health Workers (CHWs) to work alongside the clinic. In 2012, 10 CHWs were trained across 10 farms to work both with the mobile clinic and independently to test for malaria, support HIV treatment, run support groups and provide basic medical care within the farm compounds.

As MSF was developing and implementing its CHW programme, the IOM, working with the Centre for Positive Care (CPC) a local non‐governmental organisation that acted as an implementing partner, trained on many of the same farms 103 farm workers as peer educators, referred to as Change Agents. This was part of the organisation's Ripfumelo project, a large‐scale regional project which looked ‘to reduce HIV and TB vulnerability amongst migrants and mobile populations and the communities affected by migration’ across several areas in Southern Africa, including Musina. The primary difference between the two groups being that Change Agents educate and mobilise workers to seek care, while CHWs are able to provide some basic biomedical care.

**Figure 1:**
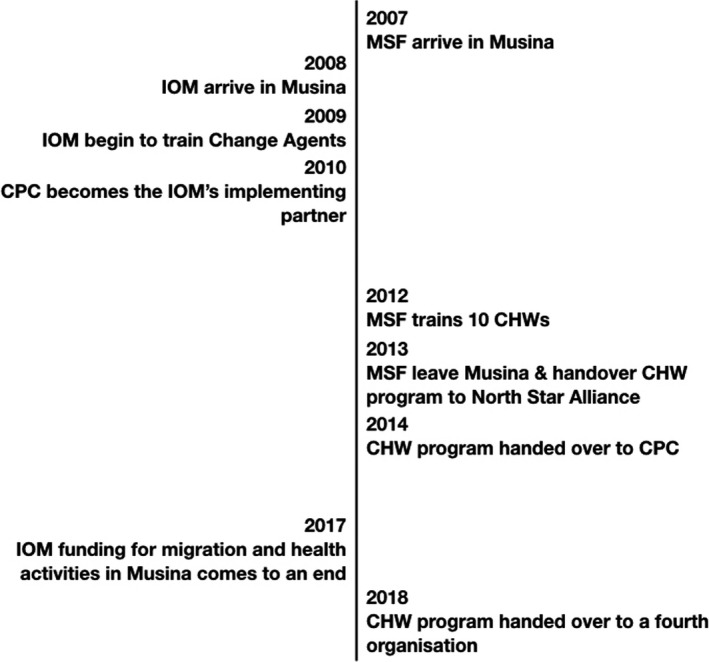
Timeline of the Change Agent and Community Health Worker programmes

Both of these interventions were envisaged as supporting the provision of biomedical care through the mobile clinic. At the height of the interventions’ success, CHWs and Change Agents were part of a robust migration‐aware response to the intricacies of healthcare access for this group of workers. However, neither MSF nor IOM could commit to sustaining the programmes for more than a few years. MSF had hoped that when they left the CHWs would be incorporated into the Department of Health. However, at the time of MSF’s exit, the Department argued that it was not able to take over the cost and the maintenance of the programme. As such, the programme was managed and funded by North Star Alliance, an international NGO that provides health services to mobile workers, for a year, after which it was handed over to the CPC, this time as an implementing partner of the Department of Health. CPC, at this time, remained an implementing partner of the IOM and involved in the training and management of Change Agents. At the end of 2017, the IOM brought an end to its migration and health related activities in the area, including funding for CPC. CPC have consequently left the area, handing the management of the CHW programme over to the local branch of a humanitarian organisation in the area, but leaving no provision for the Change Agents (see Figure 1 for a timeline of these two programmes).

This paper is based on research conducted as part of a broader project that has examined the role that non‐state actors have played in responding to migration and health in South Africa, and the longer term implications of their involvement with migrant farm workers around Musina (see Author and others, 2019). Key issues on which the research reflects have included the nature of responses by both state and non‐state actors to migration and health. This paper uses the CHWs and Change Agents to illustrate the ways in which such workers can form part of a sustainable, migration aware response to health. But the paper also demonstrates how this is undermined by both the timebound nature of non‐state interventions and the state's reluctance to incorporate community‐based healthcare workers more formally within the health system.

## 2 MATERIALS AND METHODS

Several qualitative methods were used in this research, including key informant interviews, an analysis of grey literature and observation of the mobile clinic programme.

### 2.1 Key informant interviews

A total of 79 in‐depth, key informant interviews were conducted, the specifics of which can be found in Table 1.

**Table 1:** Details of key informant interviews

Interviews conducted with	Number of interviews conducted
Agricultural sector organisations	
1 female	5
1 group (mixed)	
3 males	
Change agents	
2 females	11
2 groups (mixed)	
7 males	
Civil servants (excluding DoH Musina)	
1 female	5
1 group (mixed)	
3 males	
Community Health Workers (CHWs)	
3 females	3
Farm management	
1 female	5
2 group (mixed)	
2 males	
Farm workers	
18 females	32
1 group (all female)	
13 males	
Non‐governmental organisations (local and international)	
6 females	11
5 males	
Mobile clinic staff	
3 females	4
1 group (mixed)	
Other	
2 females	3
1 group (all male)	
Total	
37 females	79
9 groups	
33 males	

To understand workers’ experiences of the interventions, interviews were conducted across two farms on which the interventions had been implemented and where farm management were willing for researchers to conduct interviews during work hours. Most of these interviews were conducted by two research assistants who had received training on conducting interviews with farm workers, and the ethics thereof, and were able to conduct interviews in ChiShona, the language of choice for many of the farm workers. Audio recordings of interviews that were conducted in ChiShona were sent to a translator, who both translated and transcribed the interviews into English for analysis by the author.

Individuals were approached as they stopped work for lunch, waited for the mobile clinic to set up or leave, or were introduced to the researchers by others who had already been interviewed. Informed consent was sought prior to interviews commencing. Interviewees were asked to provide either written or verbal consent to being interviewed, as well as their consent to the use of a voice recorder. Of the farm workers, including Change Agents, interviewed, only two were South African. The remaining 41 were Zimbabwean nationals. Group interviews were conducted where the initial interviewee contacted indicated that their colleague would have information or insights of relevance.

Data from interviews was thematically analysed using Dedoose 8.2.14. 74 codes were identified through the initial analysis of the data, and these were then examined in relation to one another as themes.

### 2.2 Observation

It proved challenging to formally interview many of the Department of Health employed healthcare professionals who work on the mobile clinic given the constraints of their work. As such, observation of the mobile clinic was undertaken between May and July 2017. Time was spent in the mobile clinic offices (specifically in the morning as the nurses prepared to leave for the day), travelling with the mobile clinics and observing how the healthcare workers related to workers when they arrived on the farms. Observations and informal conversations were recorded as field notes, which were subsequently thematically analysed. No treatment or care itself was observed.

### 2.3 Grey literature

In addition, to supplement a review of published literature, a thematic analysis of 76 documents—including policy directives, meeting minutes, memos, project reports, policy documents and emails relating to the projects—was undertaken. By‐and‐large, these documents were collected as interviews took place and participants indicated that a particular document might be of interest or use. Documents were thematically analysed in conjunction with interview data to triangulate information and provide details that may have been forgotten by participants. Use of these data were limited however, as key informants had to be relied upon to send the author documents that they had deemed sufficiently relevant to keep.

The details of this study were reviewed and approved by the University of the Witwatersrand Non‐Medical Research Ethics Committee (REC). Ethical clearance was given under protocol H16/08/10.

## 3 RESULTS

In this section, the two programmes are presented together to highlight their differences, as well as the ways in which the two workforces interacted and continue to interact against a fragmented policy and programmatic landscape.

### 3.1 Improving access to care

While the two groups were conceptualised and trained as different kinds of community‐based healthcare workers, central to both programmes was the expectation that these interventions would improve the knowledge of and access to healthcare that migrant farm workers had.

As MSF’s hope had been that the CHWs would be incorporated into the Department of Health when MSF left, the original cohort of CHWs trained by MSF were trained using accredited state curricula for lay HIV counsellors (iNGO_06). However, since MSF has left, the training of CHWs has become ad hoc. None of the CHWs interviewed as part of this research were part of the original cohort of MSF‐trained CHWs, and none indicated having undergone state accredited training.

The experiences of the CHW programme are in some respects similar across both farms; CHWs are regarded as important sources of care and expertise, specifically around HIV/AIDS. As one farm worker indicated:

The Community Health Worker advises people that, if you [have] been found to be with these diseases (HIV and AIDS) this does not mean that you no longer have life, but that you are able to live life after contracting this disease. The Community Health Worker also advises people that if you have contracted this disease, you must take your treatment in a proper way advised for you to live longer. (MFW_LM_101)

The Change Agents, on the other hand, were trained to act as community leaders, facilitate dialogue, educate farm workers on issues of healthcare, dispense condoms, and plan weekend activities so ‘that farm workers use their free time in sports rather than engaging in unprotected sexual activities and abusing alcohol and drugs’ (IOM_10). Given this broader range of potential roles that Change Agents could and can play, these individuals are not exclusively regarded as healthcare workers by community members.

Most farm workers interviewed reported positive experiences of both CHWs and Change Agents, and importantly that they know who these individuals are and the work that they do. However, across the two farms, three farm workers reported that they would only be able to identify the CHW by face, rather than by name, and three reported that they were unaware of the CHW. While these are not large numbers, they do highlight the limitations of community‐based workers: even within a confined farm there are those farm workers who, for whatever reason, are beyond the CHW’s reach.

### 3.2 Motivations and status

While the experiences that farm workers had of the CHWs and Change Agents are important, the experiences that these individuals had and have of being part of the programmes and of trying to fulfil their roles in the wake of MSF and the IOM’s departures emerged as a central theme in this research.

Part of the initial success of the Change Agent programme can be attributed to the fact that workers saw the process of becoming a Change Agent as personally beneficial. Primarily it appears that the opportunity to be trained and receive a certificate to such effect fitted within what individuals saw as their broader life trajectories and created a sense of purpose outside of their work on the farm:

As for me, I decided to be a Change Agent because I was a person who had a course for teaching people about life and how people should manage themselves, whilst at the same time being very careful in safeguarding their lives as well as issues that are related to hygiene … Plus, at that time, there were many diseases at this farm and people (farm workers) were also being (sexually) abused (by senior employees). (CA_EG_101)

In addition, both CHWs and Change Agents have an elevated status within the compound and farm as a result of their involvement in the programmes. CHWs, in particular, have currency with figures of authority, as one reflects:

When I fall sick, I call the ambulance. I have the telephone numbers for the ambulance. So, I call them by saying to them, please I am sick here. I am not feeling well. My illness will also worry the ambulance people as they will say, among themselves ‘oh no, our CHW has fallen sick’. They will then come to me and attend me … Even the police, they know about us. If we have a problem concerning the police, I call the police directly, myself … Even the white people (farm management) here, know that, if they see me among workers in the farm fields, they know what my duties are, among their workers. (CHW_03)

As noted here, CHWs in the area and state service providers, including mobile clinic staff, developed a good working relationship, which—at the time of writing in May 2019—continues to facilitate the access that farm workers have to care. Reflecting on the importance of CHWs for the mobile clinic, one nurse explained:

Most of the time when you go there, you’re trying to trace the patient, no one knows the patient. At least if you have the CHW, they’ll look for them, they’ll track the patient for us, because they stay there and know everyone. Even if someone is sick, they’re there and to call the ambulance … maybe there’s something during the week and we didn’t go, we contact them, the CHW people there … we call them and say ‘we cannot make it on this day, but we can come on this date’. (DoH_02)

Similarly, through the programme, a relationship was established between the Change Agents and the mobile clinic staff. Like the CHWs, they are able to contact emergency medical services and are now able to advise workers what services the mobile clinic is able to provide. In addition, sporting events and drama groups organised by the Change Agents have also been used as opportunities for the mobile clinic to visit and provide voluntary counselling and testing (VCT).

Across the two farms there is however one salient difference in the CHW’s role and status. On one of the farms, the CHW is very clearly regarded as an important part of farm life. She grew up on the farm and is married to the head Change Agent (and in some interviews quite explicitly referred to as ‘The wife of Sizwe’ (pseudonym)), who is a senior employee and has a closer relationship with farm management than many of the other workers. In one interview referred to by a farm worker as ‘our leader’ (MFW_EG_103), she is very present on the farm and the lives of the workers, describing herself as motivated to fulfil her role as CHW, ‘a full‐time job’.

On the second farm, however, the importance of the role of the CHW is acknowledged, but the CHW is regarded with some frustration and irritation, as she is often absent from the farm:

The problem that we have is that, she cannot be found easily … If you are lucky to find her, she can help you. (MFW_EG_201)

The role that this CHW plays on this farm is markedly different from the previous example. Here, the CHW indicates that while she has a room on the farm, this is not her home, and her role is to be present every second week—coinciding with the mobile clinic visits. When interviewed, this CHW often deferred to Change Agents and indicated that, in her opinion, the role of the CHW and Change Agents is ‘the same’ (CHW_02). Unlike the CHW on the first farm, there is little indication that she feels particularly motivated or invested in her role and life on the farm. As a reflection of this, when farm workers on this farm were asked about the CHW, they often referred to one of the Change Agents. Here Change Agents were more heavily relied upon, in some instances to fulfil the duties of the CHW:

the Change Agents that I had known of, they used to do this, if a person is injured at the soccer match, they would somehow put a bandage on this injured person or give this person anything that they had, that could stop the pain. I have not yet seen this being done by a community health worker. What I have seen is, the Community Health Worker, shouting in the compound saying, we have the mobile clinic today, please come. This is what I have seen happening in this compound. (MFW_LM_204)

### 3.3 Frustrations and insecurity

The frustrations expressed in relation to the CHW on the second farm are part of a broader set of frustrations that both Change Agents and CHWs expressed during the research.

Although the Change Agents continue to have some status within the community, the benefits that the Change Agents derive from their volunteerism have been limited by the exit of the IOM and the CPC from the area. The IOM clearly imagined the Change Agents as being self‐sufficient upon their exit. However, without external support, many Change Agents are despondent and frustrated by their responsibilities. The general inability of the Change Agents to organise activities that were once part of compound life is a source of frustration, and seen as a direct consequence of the IOM and the CPC no longer having funding:

We used to have about ten teams. I used to have meetings … I used to be given soccer balls … Right now, I am not able to get these things because I am no longer in contact with [CPC employee]. [He] as an individual, I sometimes get hold of him, but he no longer has contact with those sponsors who used to sponsor him, and now, he has nothing to give us. (CA_EG_201)

This frustration spills over into resentment over the lack of more formal recognition of their work. While Change Agents acknowledge that they signed up as volunteers, the apparent promise of compensation at some future point appears to have been a motivating factor. CPC, for their part, acknowledge this expectation, but express the view that Change Agents should have become formalised within and compensated through farm structures, although there is no evidence that this was ever discussed with farm management.

However, while CHWs are remunerated for their work, they remain financially insecure. CHWs are paid their monthly stipend through whichever organisation is acting as the state's implementing partner at the time. However, contracts between the state and these NGOs need to be renewed annually. Regular delays in this process lead to delays in the payment of CHW stipends. For example, in 2017, the CHWs around Musina were not paid until July as CPC waited for their contract with the Department of Health to be renewed. In addition, following MSF’s departure in 2013, there appears to have been very little follow‐up or consistent support and supervision from the organisations that have managed the programme. Although the CPC, for example, claims to have visited the farms regularly, and expected monthly reports directly from the CHWs, reports indicate that the organisation did not visit the farms at all during the last year (2017) of their time in Musina. In July 2018, research participants indicated that the organisation now managing the programme have yet to engage with the CHWs.

Regardless of this lack of support and the irregularities around remuneration, however, the fact that CHWs are remunerated while Change Agents are not has become a source of tension on the second farm:

She (the CHW) is earning, but I do not know how much. We are not allowed to give people medicines, but we are allowed to test people. She tests people and we also test people; this is where we do the same thing. However, she is the one who provides us with the testing equipment. The other difference is that, [she] as a community health worker, she is paid. She is earning, but I do not know how much money is it … at this farm, we have not yet been paid but we hear through rumours, that on other farms … the rumours are saying that, the volunteers are earning. They are not paid monthly or annually, but these people are happy to attend the meetings because, at times, they are given R600 or R1000 (between $42–$70 at the time of writing) per volunteer. But as for us, we have not yet received anything! (CA_EG_203)

The structural differences in the programmes, specifically around remuneration, pose a barrier to the two cadre of workforce happily co‐existing. When additional social factors exist, as on the first farm, this barrier is overcome. However, in the absence of these factors, as on the second farm, tension is a reality.

## 4 DISCUSSION

This paper fits within the broader literature in South Africa, as well as other LMIC contexts, about the sustainability of community‐based health worker programmes.

What these two examples show is that such programmes have the potential to be migration aware; within migrant communities, community‐based healthcare workers can be migrants themselves and training can include awareness about migration as a determinant of health (Vearey et al., 2017). In addition, regardless of their migrant status, workers enthusiastically took part in these programmes and, by‐and‐large, felt that the programmes had positively affected the health and well‐being of their community. However, the sustainability of the programmes has been undermined as, reflecting much of the literature, the key ‘software’ underpinning the efficacy of these cadre of workforce—motivation and supervision—as well as their integration within the broader health system have been neglected by the state (Gilson, 2006; Kok et al., 2017; Sheikh, George, & Gilson, 2014).

While the disparity in financial remuneration between the CHWs and the Change Agents emerged as a key tension in this case, both the research presented here, and the literature highlight that financial remuneration is not the sole motivation that individuals have for becoming community‐based healthcare workers (Akintola, 2011; Greenspan et al., 2013; Kidman, Nice, Taylor, & Thurman, 2014; Mwai et al., 2013; Pallas et al., 2013). A desire to learn, as well as to improve the lives of loved ones and the general health and well‐being of the community, are also key factors that motivate individuals to participate in such programmes. However, as Greenspan et al note (Greenspan et al., 2013), a ‘strong volunteer spirit … does not preclude a desire for financial rewards’. As many CHWs and Change Agents saw these programmes as an opportunity to up‐skill and improve their status within the community, the lack of continued engagement by and support from external actors is keenly felt. This echoes findings by Akintola (2011) and Suri *et al.*, with the latter highlighting the importance of supervisors and supervision in such programmes:

For community‐based programs to be successful, issues of sustainability must be addressed. Specific attention should be paid to the motivation of CHWs, such as those relating to cross‐sector resentment, individual financial stresses, and lack of emotional support. CHW program supervisors and administrators must consider the full gamut of motivating and demotivating factors in planning ongoing support.’ (2007)

The lack of supervision and integration of the programmes within the broader state healthcare system are two additional factors that undermine the sustainability of these programmes. Supervision and integration have both been identified as key enabling factors or barriers to the sustainability of community‐based programmes (Assegaai & Schneider, 2019; Mwai et al., 2013; Pallas et al., 2013). Kok et al. (2017) argue that community‐based workers need to be seen as ‘social actors’; that trusting relationships with both the communities that they serve and the healthcare system are pivotal to their efficacy and sustainability. The role that the former plays is illustrated here by the different responses to the CHWs on the two farms, which also highlight the importance of ‘community fit’ for sustainability (Pallas et al., 2013) and perhaps the lack of care that was given to ‘community fit’ in the selection of the CHW on the second farm.

Gilson and others highlight the importance of ‘workplace trust’ in health systems (Gilson, 2006; Gilson, Palmer, & Schneider, 2005). This research indicates that community‐based healthcare workers can have a good working relationship and trust one part of the health system, in this case the mobile clinic staff and local emergency medical services, while simultaneously not being integrated into or trusting the broader structures of the health system. Research on the relationship between community‐based healthcare workers who are able to provide some biomedical care and nurses indicates that nurses often feel threated by these workers and are prone to enforcing professional hierarchies in counter‐productive ways when forced to work together (van Ginneken et al., 2010; Schneider et al., 2008). The good working relationship between the CHWs and nurses documented here contradicts much of this. This may be because in this case CHWs are isolated on the farms, and, as such, have a very clearly demarcated role—providing support and care to farm workers between mobile clinic visits. Consequently, they are of no direct threat to the nurses who leave the farm after each visit. Regardless, trust in the broader health system is limited, and may in fact be compounded by the fact that mobile clinic staff themselves indicate little trust in the broader Department of Health, the nuances of which are described in de Gruchy and Kapilashrami (2019).

Finally, theories of sustainability point to the importance of integrating interventions into broader structures (Schell et al., 2013; Shigayeva & Coker, 2015). Here, implementing organisations were unable to secure this integration prior to their departure, due to the state's fragmented interest in and response to such interventions, characterised by a lack of sufficient attention to or support for this workforce (Daniels et al., 2012; van Ginneken et al., 2010; Schneider et al., 2008).

This paper shows how community‐based healthcare workers can, and do, play an important role in health systems. However, as it makes clear, within the South African system, the fragmented approach to these workers undermines this potential.

The findings from this research support the call for migration and health to be prioritised within policy and programmatic responses to well‐being (Vearey, 2018; Vearey et al., 2017; Wickramage & Annunziata, 2018), and highlight the role that community‐based healthcare workers can play within this. However, it raises important questions about the development of these cadre of workforce within a context in which factors enabling their sustainability are limited. As such, ensuring that the ways in which migration and health are included in policy making are sustainable emerges as a necessary element to be included in global calls.

## CONFLICT OF INTEREST

There are no conflicts of interest to be declared.
